# A Review of Authorship Inflation and Multicenter Collaboration Trends in Orthopedic, Medical, and Surgical Journals Over the Last 60 Years

**DOI:** 10.7759/cureus.66107

**Published:** 2024-08-04

**Authors:** Hong-Him Dickson Chau, Zhi-Wei Jonathan Gan, Hamid Rahmahtullah Bin Abd Razak, John Carson Allen, Suang-Bee Joyce Koh, Tet-Sen Howe

**Affiliations:** 1 Orthopedic Surgery, Sengkang General Hospital, Sengkang, SGP; 2 Orthopedic Surgery, Changi General Hospital, Simei, SGP; 3 Orthopedics, Sengkang General Hospital, Sengkang, SGP; 4 Biostatics, Duke-National University of Singapore Medical School, Queenstown, SGP; 5 Orthopedic Surgery, Singapore General Hospital, Bukit Merah, SGP

**Keywords:** journals, multicenter studies, trends, publication, authorship

## Abstract

Over the past six decades, authorship numbers in academic publications have increased significantly, a phenomenon known as authorship inflation. This study aims to analyze comparative authorship trends and the influence of multicenter collaborations across major orthopedic, medical, and surgical journals.

We reviewed metadata from *The New England Journal of Medicine* (NEJM), *Annals of Surgery* (AS), and *The Journal of Bone and Joint Surgery* (JBJS) from January 1, 1960, to December 31, 2019. The number of authors per publication, the prevalence of multicenter studies, and their correlation were analyzed. Data was visualized using heat maps and box plots, and trends were statistically tested using the Jonckheere-Terpstra, Mann-Kendall, and generalized linear mixed models (GLMMs).

A total of 73,062 articles were analyzed, with 1,190 articles identified as originating from multicenter studies. The number of multicenter trials was found to have increased significantly over time (p < 0.001), plateauing in NEJM but continuing to rise in JBJS and AS. There was a significant increase in authorship numbers per publication over time, across all journals (p < 0.0001). There was a significant statistical correlation (p < 0.0001) as indicated by the coefficient of determination (r^2^), for the association between the proportion of publications with >10 authors and the proportion of multicenter publications across all three journals.

Authorship inflation in academic publishing may be attributable to the rise in multicenter collaborations. The rate of increase in authorship was more pronounced in medical and surgical journals compared to orthopedic journals, reflecting differing trends across specialties. These findings highlight the evolving nature of research collaboration and authorship practices in academic publishing.

## Introduction and background

Research manuscripts may be thought of as the currency by which authors' contributions are measured. The number of co-authors credited per published article in peer-reviewed journals has crept upward over time, leading to authorship inflation.

In the early years (1928) of *The New England Journal of Medicine (*NEJM), single-author papers were by far the most common, comprising 78% of articles published. By 1968, the number of single-author papers comprised just 3.1% of the total published [1].

To determine the trend of authorship inflation in peer-reviewed journals and its relationship to multicenter collaborations, we reviewed the metadata of every PubMed-indexed citation from a high-impact medical, general surgical, and orthopedic journal from January 1960 to December 2020 to compare authorship trends across various major specialty journals.

We aim to determine the extent of authorship inflation (inflation in the number of authors per publication) in publications and test our hypothesis that the rate of increase in authorship per publication is correlated with the rate of increase in multicenter collaborations.

## Review

Method

We tabulated every PubMed-indexed citation from January 1, 1960, to December 31, 2019, from NEJM (impact factor: 74.699), *Annals of Surgery* (AS, impact factor: 9.203), and *The Journal of Bone and Joint Surgery* (JBJS, impact factor: 4.578). The following metadata was extracted and compiled into spreadsheets in Microsoft Excel 2019: “PMID,” “Journal ID,” “Article Title,” “Publication Type,” “Journal: Year,” “Authors (Last, Initials),” and “Abstract.” Duplicate entries were removed, and “Journal ID” was cross-checked to confirm publication. We excluded obituaries, tributes, memoriams, portraits, public addresses, letters, comments, and errata.

The remaining citations were then reviewed for authorship by analyzing the “All Authors (Last, Initials)” and “Journal: Year” metadata and the number of authors per annum was tabulated (n = 1-10, >10). A heat map for each journal was created to visualize general trends. Trends were further analyzed using boxplots, and the Jonckheere-Terpstra and Mann-Kendall statistical tests for monotonic trends.

Metadata was then combed for the specific terms “multicenter,” “multicentre,” “multi-center,” “multi-center,” and “collaboration.” Unique abstracts found to contain these terms were individually reviewed for relevance, and the true multicenter publications were tabulated as a proportion of total publications per year.

We investigated trends in the proportion of multicenter trials published per year (as a proportion of total papers published), as well as the differential trends between specialties. The GLIMMIX procedure for generalized linear mixed models (GLMMs) was performed on the data for the percentage of multicenter trials published per year to determine the presence of a directional trend if any, as well as differences in trends between journals.

The differing rates of increase of authorship between specialties were investigated. A generalized linear model analysis incorporating a gamma error distribution with a natural log (ln) link function was performed on the data of mean authors per paper over time. Terms in the model were a year (1960-2018), journal (NEJM, AS, JBJS), year × journal interaction, and year^2^ × journal interaction, where the year was analyzed as a continuous variable. Trends among the journals were characterized, allowing inferences on rates of change and comparisons of differences among journals in mean authorship over time. The SAS GLIMMIX procedure (SAS Inc., Cary, USA) was used to perform the generalized linear model analysis.

The overall trend of authorship numbers was analyzed. The number of publications in each year was categorized by the number of authors and tabulated, and heat maps were generated for each journal, using a color gradient to represent the density of publications, with the X-axis showing the years, the Y-axis showing the number of authors per publication, and the color intensity indicating the frequency of publications. The Jonckheere-Terpstra test for monotonic trends was applied. The median number of authors and interquartile ranges were calculated. The Mann-Kendall test for a monotonic trend in a time series was also applied to the “number of authors” to determine the presence of directional trends.

We considered if increasing authorship numbers were related to the increase in multicenter publications. Correlation analysis was carried out between the proportion of publications with more than 10 authors (>10 authors) and the proportion of multicenter publications using a scatter plot with a line of best fit and calculation of the coefficient of determination (r^2^) from Pearson's correlation coefficient (r).

Results

**Figure 1:**
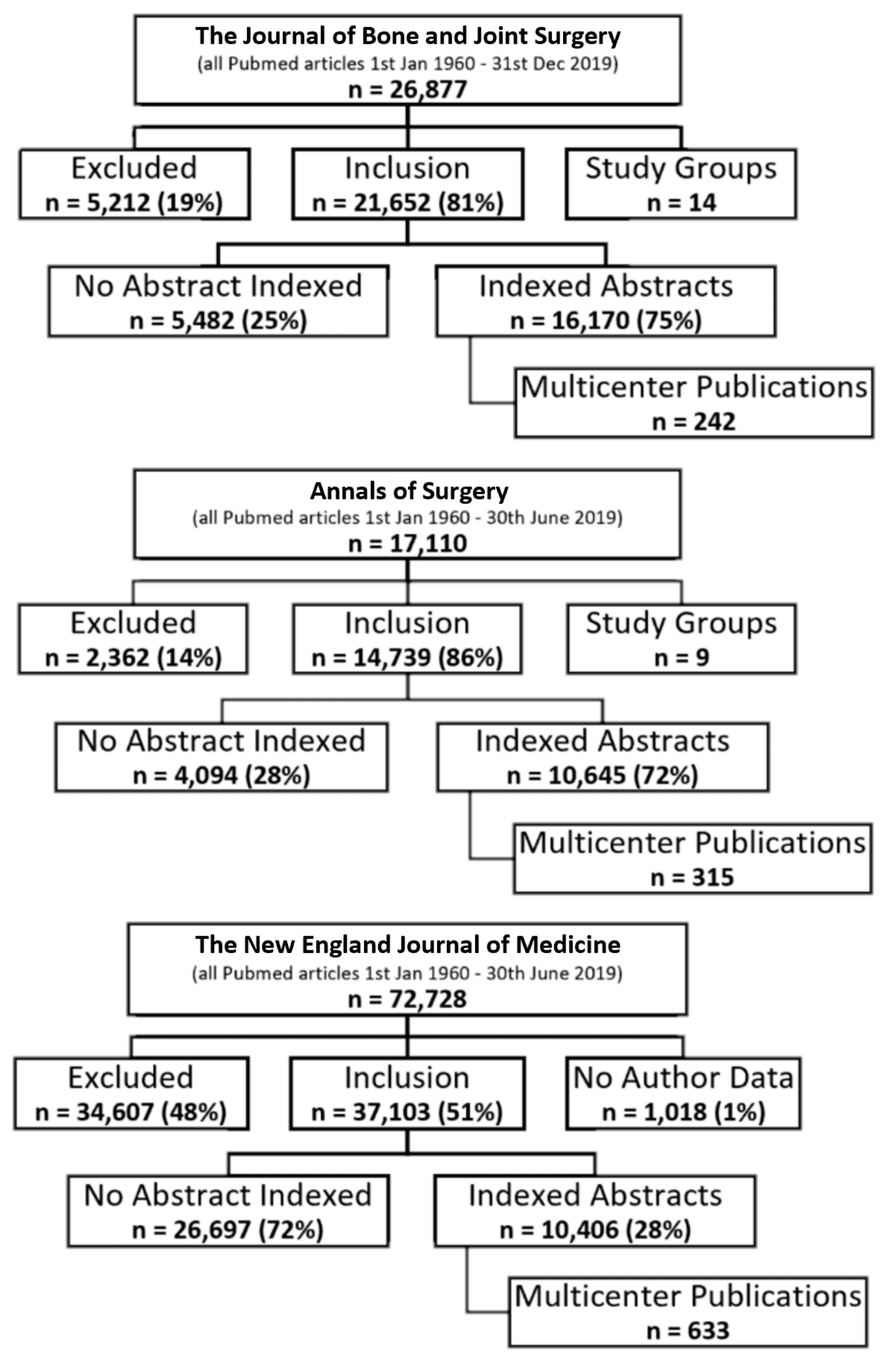
Study selection flowcharts for all three journals

The PubMed search for the specified period of January 1, 1960-December 31, 2019 returned a total of 116,715 unique articles (JBJS: n = 26,877; AS: n = 17,110; NEJM: n = 72,728) for review. From JBJS, 5,212 articles were initially excluded, with the exclusion of a further 14 articles that were submitted by study groups without authorship data. From AS, 2,362 articles were initially excluded, with the exclusion of a further 9 articles by study groups. From NEJM, 34,607 articles were initially excluded, with the exclusion of a further 1,018 articles without authorship details. The study selection flowcharts in Figure 1 illustrate this.

It is significant to note that for data from October 28, 1983, until the end of 1995, the MEDLINE article policy limited metadata to a maximum of 10 authors. These restrictions were lifted in mid-2005, and individual records could be edited to include more than 10 authors in a published article.

**Figure 2:**
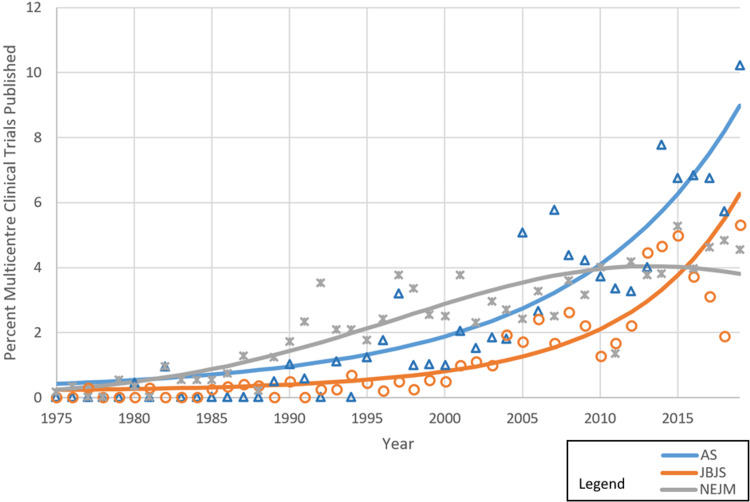
Percentage of multicenter trials published per year (as a proportion of total papers) AS: *Annals of Surgery*; JBJS: *The Journal of Bone and Joint Surgery*; NEJM: *The New England Journal of Medicine*

The proportion of multicenter trials published per year (as a proportion of total papers published), and differential trends between specialties are illustrated in Figure 2.

There were significant linear (p < 0.0001) and quadratic (p < 0.0001) increases over time in the percentage of multicenter trials published per year. The trajectories of the graphs for different journals show a differing rate of increase between journals over time. For AS and JBJS, the increase appears to be an upward curve, whereas for NEJM, the overall trend appears to have plateaued.

**Figure 3:**
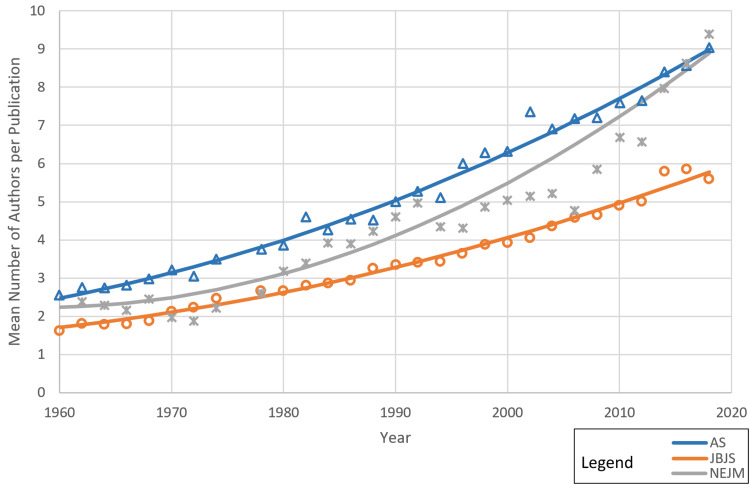
Mean number of authors per publication trended over the observed period AS: *Annals of Surgery*; JBJS: *The Journal of Bone and Joint Surgery*; NEJM: *The New England Journal of Medicine*

The differing rates in the increase of authorship between the journals from the three different specialties are illustrated in Figure 3, which shows that the trend for mean authors per paper followed an upward curve for all three journals, with second-order (accelerating) rates of increase for NEJM and AS. NEJM saw the most rapid rate of increase in authorship numbers. The increase in numbers appears to be accelerating in recent years.

Mean authorship numbers at the end of the observational time period (2018) were similar for AS and NEJM, and not significantly different (p = 0.721), with around nine authors per paper (9.0 for AS, 8.9 for NEJM), whereas JBJS saw lower rates of increase in the mean number of authors over time with significantly fewer mean authors per paper (5.7) at the end of the time period (p < 0.001).

**Figure 4:**
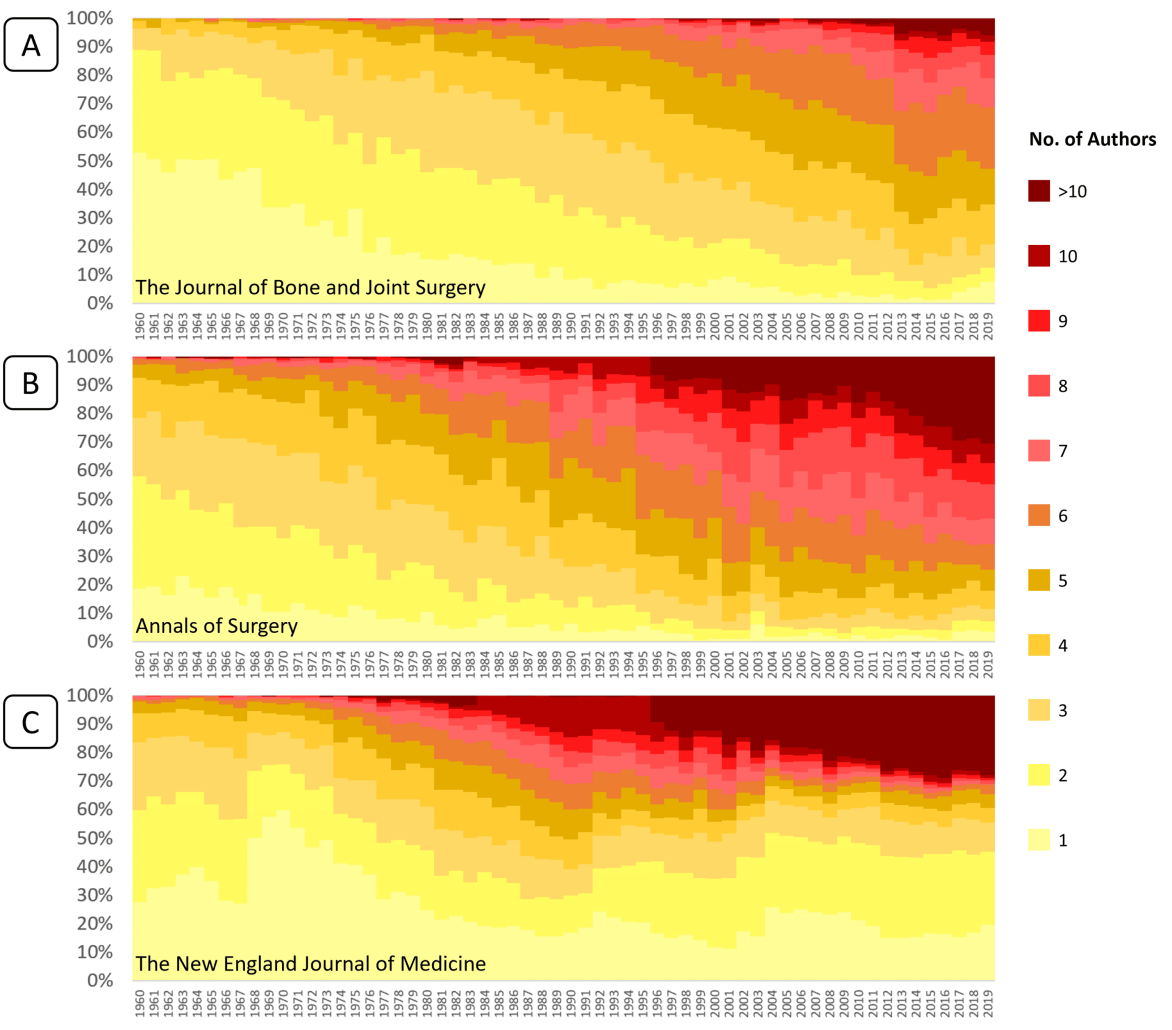
Heat maps of the percentage of authors per publication, per year Heatmap A: *The Journal of Bone and Joint Surgery* (JBJS); heatmap B: *Annals of Surgery *(AS); heatmap C: *The New England Journal of Medicine* (NEJM)

**Figure 5:**
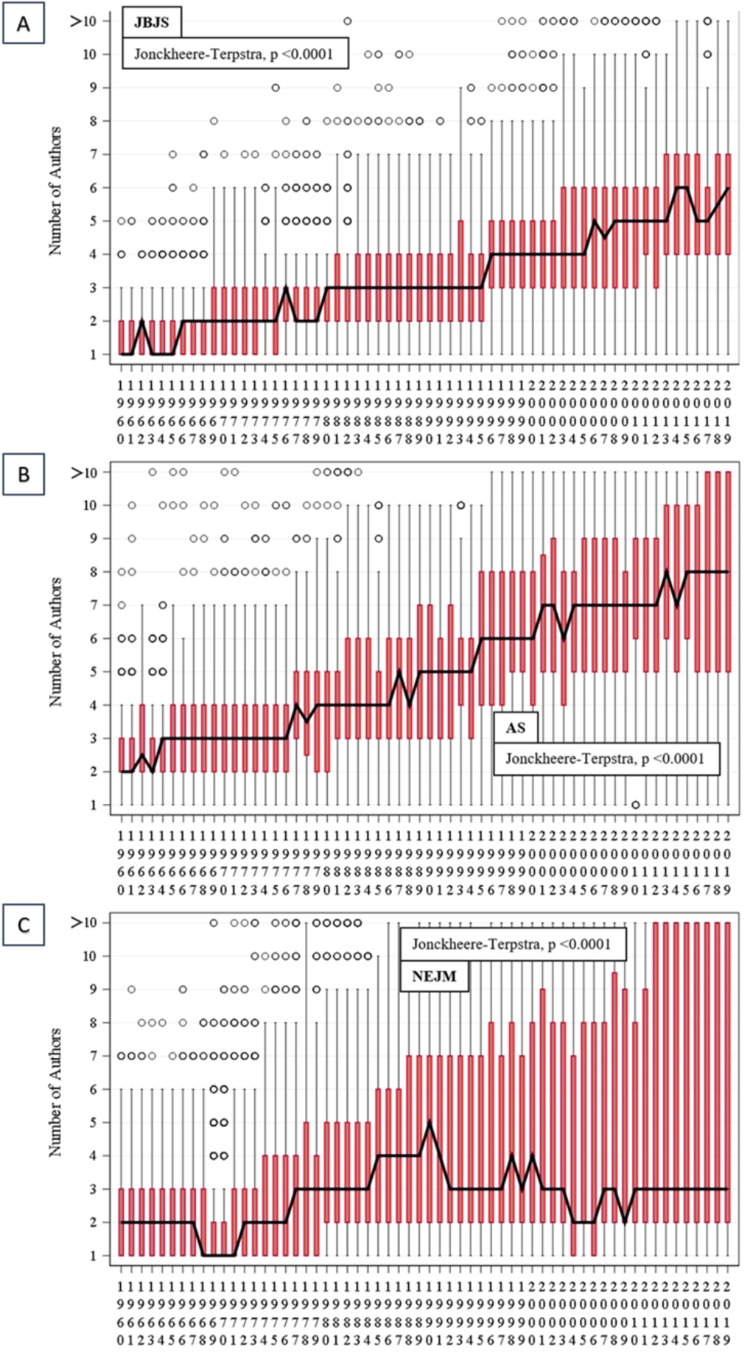
Boxplots illustrating authorship trends across the three journals Boxplot A: *The Journal of Bone and Joint Surgery* (JBJS); boxplot B: *Annals of Surgery* (AS); boxplot C: *The New England Journal of Medicine* (NEJM)

The trends in overall authorship numbers can be visualized in the heat maps illustrated in Figure 4 (the trends in JBJS are represented by heatmap A, AS is represented by heatmap B, and NEJM is represented by heatmap C), and also in the boxplots illustrated in Figure 5 (the trends in JBJS are represented by boxplot A, AS is represented by boxplot B, and NEJM is represented by boxplot C).

The number of publications in each year was categorized by the number of authors, and heat maps (Figure 4) were created for each journal over the specified period of study. The number of authors per publication increased over time, as did the proportion of publications involving >10 authors in each journal.

The Jonckheere-Terpstra statistical test for a monotonic trend was statistically significant (p < 0.0001) for each journal. Progressive increases in the median number and interquartile range of authors were observed in the publications of JBJS and AS (Figures 5A-5B). The overall median and lower quartile number of authors in NEJM (Figure 5C) publications remained relatively stable but with progressive increases in the upper quartile.

The Mann-Kendall non-parametric statistical test for a monotonic trend in a time series was also applied to the “number of authors.” Correlation values for authorship trends in JBJS and AS were 0.43 and 0.47, respectively, whilst the correlation value for NEJM was lower at 0.22. Statistically significant monotonically increasing trends (p < 0.0001) were observed for all three journals.

**Figure 6:**
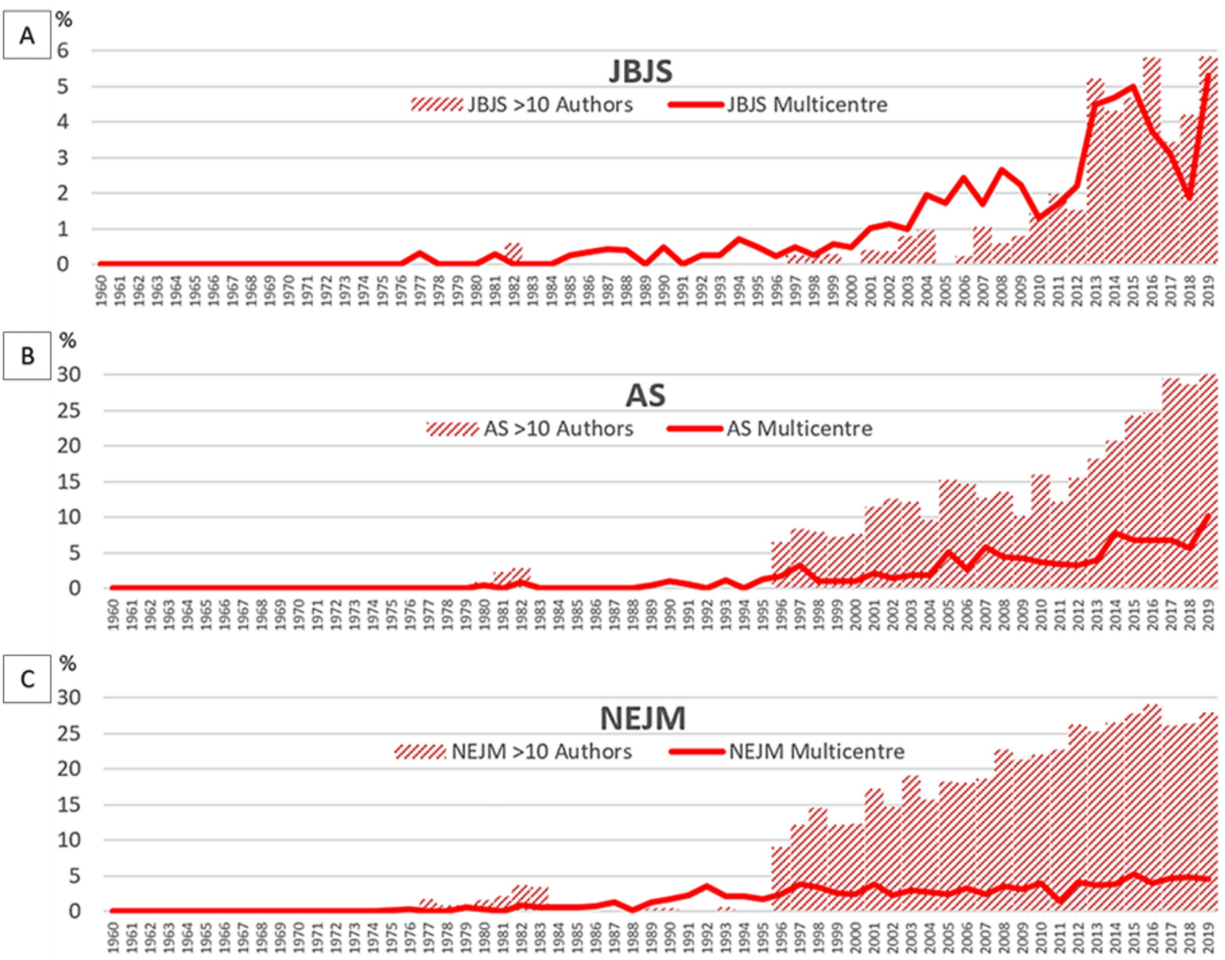
Percentage of publications >10 authors and percentage of multicenter publications across the three journals Chart A: *The Journal of Bone and Joint Surgery* (JBJS); chart B: *Annals of Surgery* (AS); chart C: *The New England Journal of Medicine *(NEJM)

The association between papers with >10 authors, and the proportion of multicenter publications were then compared, and illustrated in Figure 6 (the association between multicenter publications and publications with >10 authors in JBJS is represented by chart A, AS is represented by chart B, and NEJM is represented by chart C, respectively).

The proportion of publications with >10 authors and the proportion of multicenter publications in JBJS (Figure 6A) both increased similarly with time. Whereas in AS (Figure 6B), there was a gradual increase in the proportion of both from the 1990s, with a greater magnitude of increase in the proportion of publications involving >10 authors than the increase in the proportion of multicenter publications. There was an increase in multicenter publications in NEJM (Figure 6C) from the 1990s, which plateaued in comparison to the progressive increase in the proportion of publications with >10 authors involved.

There was a significant statistical correlation (p < 0.0001) as indicated by the coefficient of determination (r^2^), for the association between the proportion of publications with >10 authors and the proportion of multicenter publications, across all three journals. Specifically, the proportion of publications with >10 authors showed a strong correlation with the rise in multicenter studies, with r^2^ values of 0.809 for JBJS, 0.878 for AS, and 0.747 for NEJM (all p < 0.0001).

Discussion

This is the first specialty-spanning study of such scale, comprising a total of 116,715 articles published in three high-impact medical journals from different medical disciplines, over a total consecutive period of 60 years. The analysis of all available data (as opposed to the more common methodology of data sampling of 5- and 10-year intervals), allowed us to perform a comprehensive analysis of the progression of authorship trends over the last 60 years.

We acknowledge that the MEDLINE metadata limitations during the period from October 29, 1983, to the end of 1995, and we have interpreted the data with this context in mind. Limiting the analysis to the period after 1996 would eliminate metadata limitations but exclude valuable historical data. By including data from this time period, we believe our approach provides a comprehensive analysis of the evolution of authorship and collaborative practices over time. This broader perspective captures real-world influences on authorship trends while being mindful of the limitations.

More Multicenter Trials in NEJM and AS Than in JBJS

We found that over time, the percentage of multicenter trials published in the medical (NEJM) and surgical (AS) journals increased more than it did in the orthopedic journal (JBJS). This may represent a difference in the extent of collaboration between orthopedic surgeons, compared to medical specialists and general surgeons.

Brophy et al. found that orthopedic surgery literature had significantly fewer multicenter collaborative efforts than other surgical subspecialties and general medical literature [2]. They reasoned that surgeons may be more individualistic, and may be involved in fewer large drug trials (which involve many authors). Other surgical subspecialties had significantly higher proportions of collaborative studies.

Differing Rates in the Increase of Authorship Between the Three Journals From Different Specialties

Many specialties have seen their authorship rates increase, albeit at different rates. We found that authorship numbers increased with time (as illustrated in Figure 5), but it was clear that the number of authors increased differentially between specialties, with JBJS seeing a slower rate of increase than AS and NEJM.

**Figure 7:**
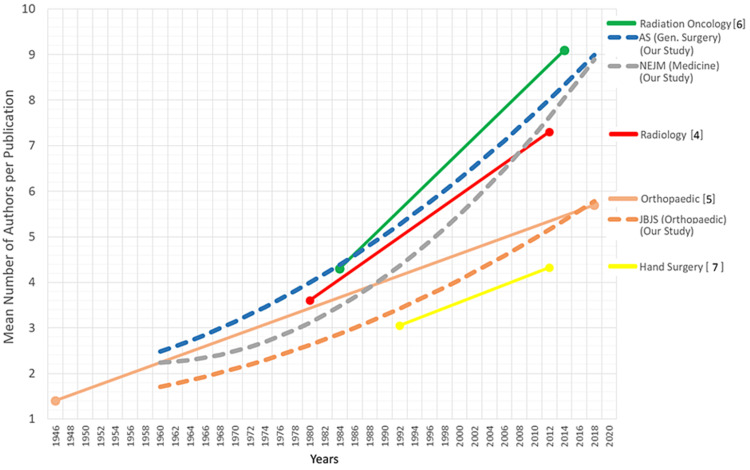
Trends in authorship numbers across specialty publications - available literature with inclusion of our study findings AS: *Annals of Surgery*; NEJM: *The New England Journal of Medicine*; JBJS: *The Journal of Bone and Joint Surgery* References: [4-7]

A literature review of other studies published showed differing rates of increases in authorship numbers between other specialties as recorded in Table 1 and illustrated in Figure 7. No clear overlying macroscopic trend was discernible (e.g., surgical vs. medical specialties). Certain specialties may have seen more advancements in knowledge, paradigm shifts in management, or benefited more from innovation, leading to an increased interest in research and increased authorship numbers.

**Table 1:** Available literature - differing trends in authorship numbers across specialties References: [3-7]

Paper	No. of articles	No. of journals surveyed	Years spanned	Earliest year	Authors per article	Latest year	Authors per article	p-value
An et al. (2018) [3]	21336	4	10	2006	5.49	2016	8.02	<0.001
Kapoor et al. (2015) [4]	682	3	43	1980	3.6	2013	7.3	<0.001
Lutnick et al. (2021) [5]	106529	10	70	1946	1.4	2019	5.7	<0.05
Ojerholm et al. (2015) [6]	2005	1	30	1984	4.3	2014	9.1	<0.001
Sugrue et al. (2015) [7]	Not stated	7	20	1993	3.05	2013	4.33	0.021

Increasing Overall Authorship Numbers

Numerous factors have been suggested to explain this trend. Positive explanations offered include the increasing complexity of research methods, increasing sophistication of research methodology, improving levels of evidence of published research, and increases in multi-department and multicenter collaboration between departments and institutions [6,8,9]. Negative factors cited include the pressure to publish by medical professionals and medical students, and gift authorship, where an author is credited with authorship despite minimal contribution [8,10].

Possible attributable causes to the authorship inflation include the increasing complexity of research, where the complexity of research methods has increased over time. We found an increase in the proportion of multicenter trials, and an association between the proportion of publications with more than 10 authors with the proportion of multicenter publications, suggesting that increases in authorship numbers are at least partly due to increasing numbers of multicenter trials. Tilak et al. had similar findings, reporting that the number of single- and multicenter randomized controlled trials (RCT) in medical publications had increased significantly from the 1960s [11]. The length of manuscripts and the number of references per manuscript can be a proxy for complexity, and these have also increased over time [3].

Another factor is the increasing collaboration and multi-disciplinary research efforts. We found that the number and proportion of multicenter trials, as well as the number of published multicenter trials, increased significantly over time. This suggests increasing collaboration, echoing existing findings [6]. While collaboration contributes to increasing authorship numbers, the proportion of multicenter trials published remains low, limiting the overall effect.

Furthermore, the overall level of evidence of published papers has improved over time, with journals in various disciplines seeing progressive increases in the percentage of level I and II studies. These have included journals in pediatric orthopedics, orthopedic surgery, sports medicine, plastic surgery, and oral and maxillofacial surgery [12-16]. The percentage of level I studies has increased significantly, from 4% in 1985 to 21% in 2005 [9]. Such studies typically have a larger number of authors, contributing to an increase in authorship.

Authorship proliferation may also be due to the increasing use of authorship and research as a yardstick by which individuals are measured, whether medical students or senior medical professionals. Authorship is sometimes required for promotion and may be tied to remuneration, with some countries offering financial incentives for publication in a high impact factor [17,18]. The publish-or-perish mindset extends to medical school. Wickramasinghe et al. noted an exponential increase in medical student publications since the 1980s, especially in psychiatry, general medicine, medical education, and oncology [19]. Candidates applying for residency often aim for publication of manuscripts in a peer-reviewed journal to improve their chances, with competitive residencies tending to attract students to research [5].

The decreasing share of case reports in favor of other study types has also likely contributed to increasing author numbers, as the modern case report typically has a maximum of 3-5 authors, a relatively small number. From the 1800s up to the mid-to-late 1970s, the case report was the most common type of journal article published in the Edinburgh Medical Journal [20]. In the 1980s, evidence-based medicine gained popularity, and the proportion of other study types published increased. These studies, such as cohort studies and case-control studies, featured larger numbers of patients [21,22]. Case reports, having significantly lower citation impact [23], fell out of favor. Case reports are often not taken into consideration by institutions and committees where promotion and tenure are concerned [8], which combined with the pressure to publish, may partly explain the decline of case reports.

Gift authorship has been suggested as another contributing factor. In 2005, McKneally introduced the concept of the “Gift Author,” who is credited with authorship despite minimal contribution [10]. Camp and Escott [8] echoed this idea of “undeserved authorship,” finding a trend of authorship inflation in orthopedic literature. Authors in other specialties have found similar trends [24]. It was found that a number of hyper-prolific authors had published, on average, more than one paper every five days, some of whom, by their own admission, would not have fulfilled objective criteria for publication [18,25]. The International Committee of Medical Journal Editors (ICJME), in 1985, introduced authorship guidelines to more clearly define the roles of authors trend and curb authorship inflation.

Limitations

Our study was purely observational and retrospective, and for practical reasons, we were limited to a quantitative analysis of metadata. As a result, we were unable to determine the most significant cause to authorship inflation.

## Conclusions

Across the journals NEJM, AS, and JBJS, there has been a steady annual increase in the number of authors per publication over the last six decades. This trend might be influenced by the rise in multicenter collaborative publications.

The rate of increase in authorship was found to be more rapid in the representative medical (NEJM) and general surgical (AS) journals over the same period, which may indicate increased research interest or other factors that warrant further exploration.
